# The Role of Formal Policy to Promote Informed Consent of Psychotropic Medications for Youth in Child Welfare Custody: A National Examination

**DOI:** 10.1007/s10488-022-01212-3

**Published:** 2022-08-06

**Authors:** Thomas I. Mackie, Ana J. Schaefer, John S. Palatucci, Laurel K. Leslie, Stephen Crystal, Michael Gusmano, Hannah E. Karpman

**Affiliations:** 1 Department of Health Policy and Management, SUNY Downstate Health Sciences University School of Public Health, 450 Clarkson Ave, Brooklyn, NY 11203 USA; 2 Rutgers School of Public Health, 683 Hoes Lane W, Piscataway, NJ 08854 USA; 3 American Board of Pediatrics, Chapel Hill, NC USA; 4 Tufts University School of Medicine, 136 Harrison Ave, Boston, MA 02111 USA; 5 Institute for Health, Health Care Policy and Aging Research, Rutgers University, 112 Paterson Street, New Brunswick, NJ 08901 USA; 6 College of Health, Lehigh University, 27 Memorial Drive, Bethlehem, PA 18015 USA; 7 Smith College, North Hampton, MA 01062 USA; 8 University of Massachusetts Chan Medical School, Shiver Center, 55 N Lake Ave, Worcester, 01655 USA

**Keywords:** Informed Consent by Minors, Classification, Legislation as Topic, Psychotropic Drugs, Child Welfare

## Abstract

Active participation of youth and surrogate decision-makers in providing informed consent and assent for mental health treatment is critical. However, the procedural elements of an informed consent process, particularly for youth in child welfare custody, are not well defined. Given calls for psychotropic medication oversight for youth in child welfare custody, this study proposes a taxonomy for the procedural elements of informed consent policies based upon formal and informal child welfare policies and then examines whether enacted state formal policies across the United States endorsed these elements. A sequential multi-method study design included: (1) semi-structured interviews with key informants (n = 58) primarily from state child welfare agencies to identify a taxonomy of procedural elements for informed consent of psychotropic medications and then (2) a legislative review of the 50 states and D.C. to characterize whether formal policies endorsed each procedural element through February 2022. Key informants reported five procedural elements in policy, including how to: (1) gather social and medical history, (2) prescribe the medication, (3) authorize its use through consent and youth assent, (4) notify relevant stakeholders, and (5) routinely review the consenting decision. Twenty-three states endorsed relevant legislation; however, only two states specified all five procedural elements. Additionally, the content of a procedural element, when included, varied substantively across policies. Further research and expert consensus are needed to set best practices and guide policymakers in setting policies to advance transparency and accountability for informed consent of mental health treatment among youth in child welfare custody.

**Supplementary Information:**

The online version contains supplementary material available at 10.1007/s10488-022-01212-3.

## Introduction

Medications to manage emotional and behavioral health problems (hereafter, “psychotropic medications”) are prescribed to increasing numbers of children in the United States (US), including more than one-quarter of the approximately 400,540 children removed from their caregiver of origin and placed into child welfare (hereafter, “youth in child welfare custody”; Crystal et al., 2009). Concerns around the safe and judicious use of psychotropic medication among youth in child welfare custody exist due to observed prescribing patterns. Studies find that the target symptoms for prescribing of specific classes of psychotropic medications (e.g., antipsychotic medications) do not align with FDA-approved indications or where the evidence base for their use is strongest (Chen et al., 2021; Christian et al., 2013; Mackie et al., 2020a, 2020b). Other concerns include youth in child welfare custody are not receiving psychosocial treatment as a first line of treatment, as well as lack of blood glucose and serum cholesterol monitoring for potential cardiometabolic side effects (Crystal et al., 2016).

In response, federal government and legislators made calls for the United States Department of Health and Human Services (HHS), state legislators, and public sector agencies to, promote psychotropic medication oversight among children in foster care (Barnett et al., 2016b). For example, the Government Accountability Office conducted multiple federal investigations, including a report entitled “Foster Children: HHS Has Taken Steps to Support States’ Oversight of Psychotropic Medication but Additional Assistance Could Further Collaboration” (Government Accountability Office, 2011; Government Accountability Office, 2017). Moreover, the enactment of two pieces of federal legislation, the Fostering Connections to Success and Increasing Adoptions Act of 2008 (P.L. 110-351; 2008) and the Child and Family Services Improvement and Innovation Act of 2011 (P.L. 112-34 2011; 2011) required Title IV-B and Title IV-E funded child welfare agencies to develop protocols for oversight of health and mental health services and psychotropic medications specifically for youth in child welfare custody, respectively. Prior research suggests that child welfare policymakers mobilized multiple types of evidence, both “global” and “local” to their respective jurisdiction, to inform their respective responses to these federal calls to provide additional psychotropic medication oversight (Hyde et al., 2016).

In response to these federal calls for additional oversight, a rapid expansion of system-level psychotropic medication oversight policies for children in child welfare custody emerged and is well documented (Mackie et al., 2017). Prior studies suggest that 45 of the 50 states and D.C. implemented at least one system-level strategy to provide psychotropic medication oversight for youth in child welfare custody by 2013. The extensive set of state actions reflect, in part, that youth in child welfare custody present a unique set of legal and ethical responsibilities for the state and/or county child welfare agencies. These agencies serve *in loco parentis* or “in place of the parent” for such youth. As a result, the state assumes unique responsibilities in ensuring oversight and a meaningful informed consent process for youth in their care.

While studies have investigated how states mobilized in response to calls for greater oversight (Hyde et al., 2016) and whether policies exist regarding informed consent of psychotropic medications for youth in child welfare custody (Naylor et al., 2007; Noonan & Miller, 2013), limited attention has been given to the procedural elements of the informed consent processes endorsed by child welfare policies and the comprehensiveness of state policies in the United States. Accordingly, this paper proposes a taxonomy for the procedural elements of an informed consent process for psychotropic medication use among youth in child welfare custody and examines whether these procedural elements are endorsed in the formal policies enacted across the United States.

### Defining Informed Consent and Assent

Informed consent is seen as a critical component of patient-centered mental health care (Braithwaite & Caplan, 2014). Informed consent is defined as a process of consent that is based on the provision of the information required for an individual to exercise fully their decision-making authority (Katz & Webb, 2016), consisting of ethical, legal, and administrative dimensions (Hall et al., 2012). Procedural elements viewed as important for informed consent vary depending on professional guidelines, but generally seek to ensure: (1) assurance of capacity for the decision-maker to provide consent, (2) provision of information in a language understandable to the decision-maker, (3) assessment of understanding of the information, including associated risks and benefits, provided to the decision-maker and (4) documentation of consent (Katz & Webb, 2016). These procedures are generally understood to provide legal protection (e.g., preventing assault and unwanted procedures), ethical assurances (e.g., protecting patient autonomy, preferences and decisions), and administrative documentation of patients’ involvement (Hall et al., 2012).

Assent aims to ensure meaningful input from the patient as an active participant in their care and that they ultimately agree to receive the treatment, even if not granted legal decision-making authority (Katz & Webb, 2016). In this process, a clinician will: (1) establish developmentally appropriate awareness of the condition with the patient, (2) create and communicate an individualized care plan relating to treatments/procedures, (3) assess the patient’s comprehension of the care plan, and (4) work with families and patients to resolve areas of conflict and determine patient’s assent to treatment (Katz & Webb, 2016; Mosca et al., 2005). At each step of the informed consent or assent process, clinicians are encouraged to present care options clearly and in a manner that is developmentally appropriate for the patient.

### Informed Consent for Youth in Child Welfare Custody

Studies of youth, caregiver, and clinicians have highlighted the need for additional practices of patient-centered care in prescribing psychotropic medications, including the informed consent process, for youth in child welfare custody (Barnett et al., 2019). For example, a systematic review of studies investigating the patient-centeredness of psychiatric care found a pervasive lack of knowledge, limited decision-making agency, and imbalanced power between patients and providers (Barnett et al., 2019). In the informed consent process specifically, youth formerly in child welfare custody, their caregivers, and provider report the need for: (1) communication, (2) comprehension, (3) youth involvement and agency, (4) stakeholder accountability, (5) consideration of the trade-offs in having a mandated authority, (6) collaboration, and (7) attention to potential treatment delays due to informed consent processes (Simmel et al., 2021).

### State Regulation of Informed Consent

In response to these concerns, formal policies have the potential to provide procedural assurance that consent is truly informed, including elements such as transparency, accountability, and the principles of shared decision-making in informed consent procedures for mental health treatment (Barnett et al., 2019; Noonan & Miller, 2013). Broadly speaking, informed consent often is supported through legal requirements that may be outlined to varying extent through statutes, regulation, and case law across the states (Ginsberg, 2010). Among these, formal policies refer to legislation and associated regulations, statutes, and administrative code that are enacted through a public process generating a legally binding statute or rule (Noonan & Miller, 2013). Formal policies may be endorsed either as laws enacted by the legislature (in the form of statutes) or rules endorsed by an administrative agency in the form of administrative code (Rinfret et al., 2018). In contrast, informal policies are typically established as “guidance documents,” consisting of non-legislative or interpretive rules and states generally do not require any notice or comment before guidance documents are issued (Noonan & Miller, 2013). Prior studies argue that formal informed consent policies-as opposed to informal ones-promote transparency, public discourse, and protection in cases of surrogate decision-making with vulnerable populations, including youth in child welfare custody (Noonan & Miller, 2013). Notably, class action litigation has generated considerable reform in mental healthcare service delivery for youth in child welfare custody, including influencing the development of formal policies to ensure accountability and transparency in mental health oversight (Oppenheim et al., 2012).

### Study Objective

This article characterizes the procedural elements of child welfare policies that endorse an informed consent process for psychotropic medications among youth in child welfare custody. We then sought to examine whether and how these procedural elements are reflected in formal state policies across the United States.

## Methods

### Study Design

We employed a sequential multi-method design. First, we conducted semi-structured interviews in 2009 and 2010 to provide an in-depth study on states’ responses to PL 110-351 and to create a taxonomy of the procedural elements of informed consent policies articulated by mid-level administrators. Qualitative methods, like those used by our research, are appropriate and frequently engaged to develop taxonomies (Patton, 2002). In this article, we use the term “taxonomy” to refer to a formal system of classification, specifically classifying procedural elements of informed consent to identify a set of common conceptual domains. We subsequently conducted a legislative review to refine and improve this taxonomy as well as characterize existing informed consent policies nationally. Collectively, this multi-method approach permitted development of a taxonomy for understanding policy approaches to informed consent for youth in child welfare custody and characterization of existing state legislation using this taxonomy. The Institutional Review Board at [institution withheld to preserve anonymity] reviewed and approved the study protocol.

### Taxonomy Development

Semi-structured interviews provided the source for taxonomy development that we then applied to the legislative analysis.

#### Sampling Framework

Our sampling framework prioritized key informants in child welfare agencies, given the responsibility of these agencies to serve *in loco parentis.* Interview data were collected from 48 states including the District of Columbia (D.C.; hereafter ‘states’) out of a possible 51 (response rate: 94.1%). While two of the 58 key informants arrived from the Governor’s Office and the State Department of Health, the remainder held a wide range of roles within child welfare, including Program Officers (n = 16), Medical or Mental Health Directors/Specialists (hereafter, “Medical Directors” or “Mental Health Directors,” respectively; n = 12), and Deputy or Associate Directors of Policy and/or Practice (n = 6), among others (n = 22).

A trained research assistant identified key informants through online searches of state websites, a search of the National Association of State Foster Care Managers website, and a search of the Child Welfare Information Gateway website. The research assistant then contacted the informant by telephone to confirm receipt of the informed consent and willingness to participate, to ensure the subject was the best available informant, and to schedule the interview. We engaged a purposive and referral sampling strategy to ensure adequate response to the questions in the interview guide. If the first contact had insufficient information to answer all the questions, we asked that person to refer us to the appropriate informant in their state. In one state, an informant responded to our interview, but did not name other informants. Partial data points are presented for that state. Our team recruited a key informant from another sector (e.g., Governor’s Office, State Department of Health) only when a policy/guideline was developed or managed by that agency.

#### Measures

The interview included 61 closed and open-ended questions with additional probes for use when necessary. The guide sought to: (1) identify the status of state policies and programs designed to provide evaluation and psychotropic medication oversight for youth in child welfare custody, (2) examine procedural elements of child welfare medication oversight initiatives, and (3) identify potential best practice models used by states to improve medication oversight for youth in foster care. The present article relies on data about the informed consent policies specifically, drawing on three questions in particular: (1) the procedural elements of formal and informal informed consent policies for psychotropic medications among youth in child welfare custody, (2) the individual(s) vested with authority to consent and/or whether youth assent for psychotropic medication use, and (3) the additional resources deployed to inform consenting and/or assenting decisions.

#### Study Procedures

The interview guide received review from an interdisciplinary research team and was subsequently pilot tested with three child welfare mid-level administrators who provided consultation on these processes*.* Following external expert review and pilot testing of the interview guide, the research team administered the interview by telephone between March 2009 and January 2010. An experienced and trained research assistant used the study guide and recorded data by hand-written notation. Our team used this notation process because the goal was to understand the status and conceptualization of policy, program, or procedures rather than more complex social phenomenon investigating the lived experience of study respondents (Bertrand et al., 1992). The average duration of the interview was approximately 60 min. No child or case-specific data was obtained during the interview.

#### Analysis

Employing the five steps of framework analyses (Srivastava & Thomson, 2009), three investigators initially identified a thematic framework for psychotropic medication oversight, including informed consent policies, among youth in child welfare custody. In this article, we summarize the findings that related specifically to informed consent policies. The research team indexed key aspects of informed consent policies, including: (1) the procedural elements described; (2) the individual with consenting authority; and (3) the resources available to support the consenting authority in decision-making. For each of the domains indexed, our team then conducted a priori and emergent codes to analyze the indexed data, using a process of thematic analysis (Braun & Clarke, 2006). For example, the research team identified the procedural elements based upon both emergent and inductive findings as well as the extant literature, specifically drawing on guidelines published by the American Academy of Child and Adolescent Psychiatry (American Academy of Child & Adolescent Psychiatry, 2015) for psychotropic medication treatment. Accordingly, our team identified procedural elements for informed consent policies that included processes to gather, prescribe, authorize, notify, and then follow-up and review the prescribing decision. The resulting taxonomy, using data from 2009 to 2010, provided the framing for the legislative review.

### Legislative Review of Formal State Policies for Informed Consent

Based on the taxonomy identified from the semi-structured interviews, we conducted a systematic legislative review to examine the extent to which the elements of the taxonomy were formalized in state laws and regulations. To identify the laws in place nationally, the systematic legislative review examined state-level statutes and regulations relating to informed consent and the administration of psychotropic medications for youth in child welfare custody across all 50 states and D.C. on or before February 1, 2022. Legislative review is an optimal approach to systematically identify and characterize the presence of a *formal policy* delineating informed consent procedures required of youth in child welfare custody (Noonan & Miller, 2013). We employed WestLaw (Thomson Reuters Corporation, New York, NY), a repository of state statute and regulations for the 50 states and D.C. to identify relevant legislation.

#### Development of Search Strategy for Legislative Extraction

We identified search terms through a preliminary literature review and included the following domains: (1) psychotropic medications, (2) informed consent and/or assent (Naylor et al., 2007), (3) children and/or youth, and (4) foster care and/or child welfare (see Appendix 1) (Noonan & Miller, 2013). Two researchers then reviewed a sample of initial results [six states] to determine appropriateness of search terms. Based on preliminary search, we added “permission” to the search terms used for the review. We then conducted full-text searches for all 50 states and D.C. using the search terms for each policy document.

#### Extraction of Legislation

Based on search terms, we initially identified 1140 regulations and statutes, including 385 statutes and 755 regulations. Two researchers reviewed all statutes and regulations to determine whether applicable to the informed consent process for psychotropic medications, and applicable to youth in child welfare custody. We considered laws to be outside the scope of our study if they did not explicitly reference youth in child welfare custody. For example, laws targeting youth in juvenile justice facilities, without any specifications for informed consent among youth in child welfare custody, were excluded. Laws were exported into an Excel spreadsheet for analysis. After applying exclusion criteria, 48 state laws remained, including 13 statutes and 35 regulations. See Appendix A for a final list of state laws in effect on or before February 2022 which were identified using Westlaw.

#### Data Analysis

Three researchers initially analyzed statutes and regulations that met criteria for inclusion from six states. Identifying a priori categories (based on the procedural elements of informed consent derived from semi-structured interviews) and emergent categories, an initial codebook was developed and received review from a developmental behavioral pediatrician, social work policy scholar, and health services researcher. Therefore, the research team triangulated the domains established from the semi-structured interviews with key elements of procedural elements identified in the legislative review; thematic areas corroborated across the respective data sources. Accordingly, a preliminary codebook was then established specifying the procedural elements and other policy characteristics of informed consent policies. After the codebook was established, two researchers systematically reviewed all extracted statutes/regulations and coded a priori (by relevant text by domain such as each of five procedural elements) and emergent (e.g., setting) domains. Although the need never arose, the protocol stated a third reviewer would be consulted in cases where the first two reviewers could not arrive at consensus.

After coding data by procedural element or policy characteristic (i.e., setting), three researchers subsequently conducted an additional emergent thematic analysis of the excerpted data for each element. Investigators read the textual data of all excerpts in an element and provided an open code to capture detailed information about each procedural element. Based on the open coded responses, we then expanded the initial codebook of procedural elements and settings to capture the “child codes,” denoting characteristics of the respective procedural element. To categorize setting, two investigators reviewed the language used to capture the setting in which a policy applied for youth in child welfare custody. The two investigators coded the setting for which a policy applied using the language of the legislation itself wherever possible, resulting in the list of settings identified in the results. At least two members of the research team reviewed all codes. We then assessed the relative frequency and variation of procedural elements in informed consent policies and the content of these policies to determine the frequency and heterogeneity in reporting of procedural elements for informed consent.

## Results

### A Taxonomy of Procedural Elements for Informed Consent among Youth in Child Welfare Custody

For youth in child welfare custody, respondents articulated the potential for five procedural elements of an informed consent policy. As defined in Table 1, the five procedural elements of an informed consent policy included specification of how to: (1) gather the social and medical history of the youth in child welfare custody, (2) specify the activities required to inform the decision to prescribe (or not to prescribe), (3) authorize (or not authorize) the psychotropic medication(s) prescribed through consent and/or youth assent, (4) notify relevant stakeholders, and (5) review the informed consent decision for psychotropic medication use for youth in child welfare custody. Table 1 provides the definition for each of these procedural elements and the operational definitions engaged in the legislative review conducted. Description of the extent to which these elements were applied in the reviewed legislation concludes the results.

**Table 1:** Procedural elements of informed consent policies

	Gather	Prescribe	Authorize	Notify	Review
Definition	The relevant stakeholders gather the medical and social history (when available) of the youth, the presenting emotional or behavioral problem(s), and the treatment plan	The clinician and other stakeholders at the clinical encounter then choose to *prescribe (or not prescribe)* psychotropic medication as a course of treatment	In some states, a third party *authorizes (or does not authorize)* the use of medications. A third party may be the caregiver of origin, the foster parent, case worker, court system, or a medical expert(s) internal or external to the child welfare system. Additionally, authorization may not occur in cases in which assent is required. Assent provides the ability for youth to refuse the medication	Relevant stakeholders are *notified* of the decision to begin psychotropic medications. Notification might include any of the following information: name of the medication(s) administration details; targeted symptoms; and risks, benefits, and side effects	The consenting authority is set to revisit the prior informed consent decision regarding the use of psychotropic medications after a particular window of time or under specific circumstances
Operation Definition for Legislative Review	Policies may require gathering information prior to the clinical encounter to inform the decision to prescribe and authorize a psychotropic medication; information may be gathered from specific collaterals or records documenting medical and/or social history	Policies for informed consent may require specific actions to occur at the clinical encounter where prescribing psychotropic medication occurs	Policies vary in consenting authority designated by policy, as well as whether documentation is required and whether the youth have the right of refusal	Policies may require notification of consenting decision to specific stakeholders	Policies may require that the consenting authority revisit the prior decision for authorization within a specified timeframe or under specific circumstances
Illustrative Example from Policy	“Information regarding the rationale for the proposed medication, provided in the context of past and current treatment efforts, is provided to the court. This information shall include, but not be limited to, information on other pharmacological and nonpharmacological treatments that have been used and the minor’s response to those treatments.” (West’s Ann.Cal.Welf. & Inst.Code § 739.5)	“All children and youth must receive a diagnostic assessment prior to starting a psychotropic medication.” (89 Ill. Adm. Code 325 App. A 325)	“The facility shall cooperate with the contracting entity and, where applicable, the CSSD to obtain written consent from the resident’s parent(s) or guardian(s), or court order, before administering psychotropic medications.” (D.C. Mun. Regs. Tit. 29, § 6264)	“The child’s parent(s)/guardian should be notified regarding the recommended medication, unless the parent’s parental rights have been terminated.” (MS ADC 18–6:1.D-VII)	“An annual review of psychotropic medications, by an individual other than the prescriber when: (A) A child or young adult has more than two prescriptions for psychotropic medications; or (B) A child under six years of age has a prescription for psychotropic medication.” (OAR 413–070-0430)

#### Legislative Review

**Fig. 1:**
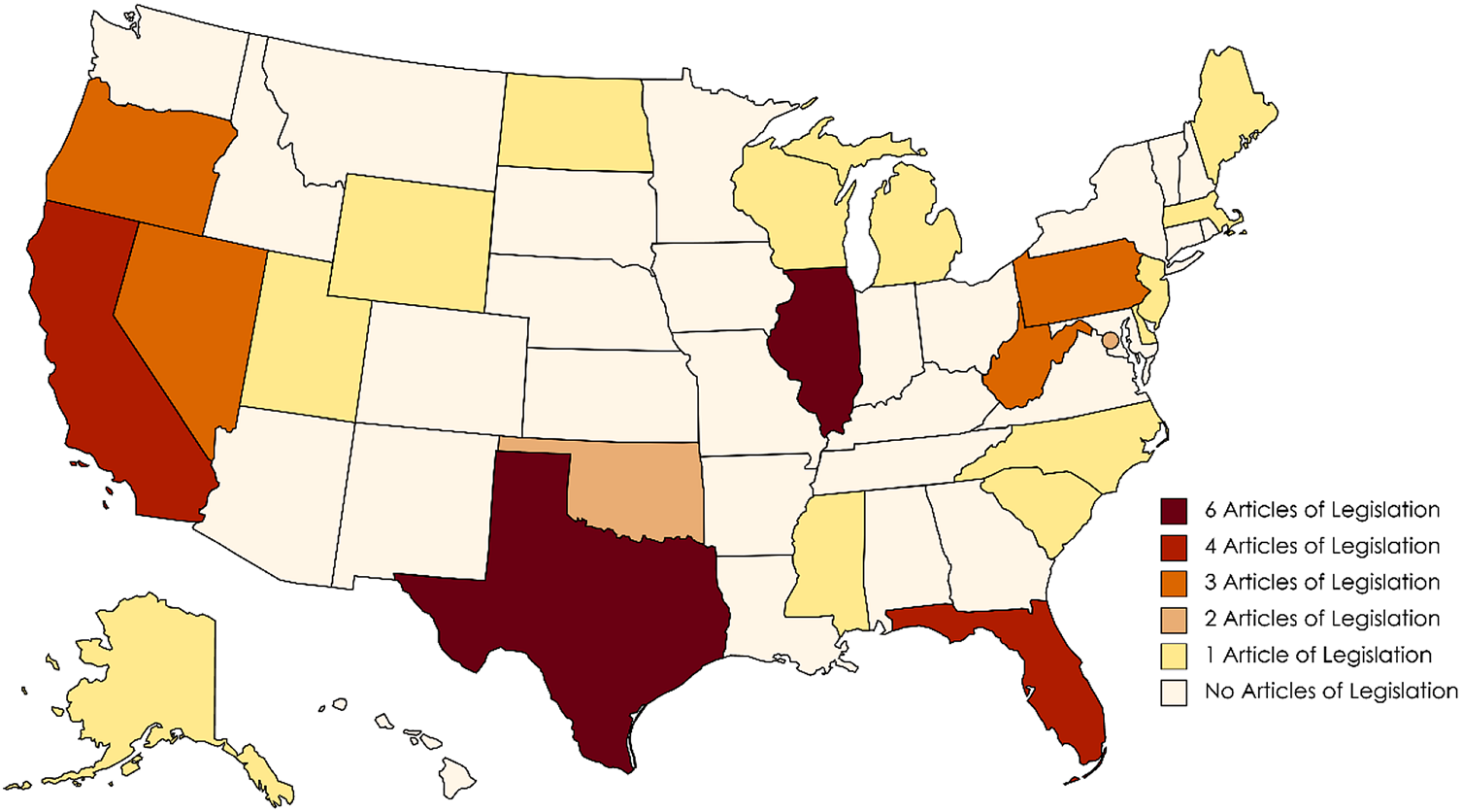
Number of legislative articles specifying informed consent for psychotropic medication use to youth in child welfare custody, by State

Once our taxonomy of procedural elements for an informed consent policy was identified, we used this taxonomy to classify the policies identified through a legislative review. Across the 50 states and D.C., 23 states (including D.C.) had at least one statute or regulation that specified a procedural element of informed consent. Figure 1 illustrates the location of states nationally having endorsed policies, specifically detailing the number of articles of legislation provided. Twenty-three states (including D.C.) had enacted 48 pieces of legislation, ranging from one to six articles per state. Enacted articles were endorsed between 1974 and 2022.

Of the 23 states that had enacted at least one legislative article, only two states (OR, CA) specified all five procedural elements of informed consent. All 23 states specified the process for authorizing the use of psychotropic medication, including designation of the individual with authority to consent for youth in child welfare custody. Specification of parameters for prescribing the psychotropic medication for youth in child welfare custody and gathering the medical and social history of the youth were the second and third most frequently specified elements, endorsed by policies in 18 and 13 of the states, respectively. Six states enacted policies that specified who would be notified of the consenting decisions. In six states, the policies specified a process for the authorizing individual to revisit the informed consent decision at a later time. Table 2 provides a summary of the procedural elements endorsed by informed consent policies across the 23 states.

**Table 2:** Overview of procedural elements for informed consent policies, by State

State	Gather	Prescribe	Authorize	Notify	Review
Alaska (AK)		X	X		
California (CA)	X	X	X	X	X
Delaware (DE)			X		X
District of Columbia (D.C.)	X	X	X		
Florida (FL)	X	X	X	X	
Illinois (IL)	X	X	X		X
Massachusetts (MA)^a^			X		
Maine (ME)		X	X		X
Michigan (MI)			X		
Mississippi (MS)	X	X	X	X	
New Jersey (NJ)	X	X	X		
Nevada (NV)	X	X	X		X
North Carolina (NC)			X	X	
North Dakota (ND)	X	X	X		
Oklahoma (OK)	X		X	X	
Oregon (OR)	X	X	X	X	X
Pennsylvania (PA)		X	X		
South Carolina (SC)		X	X		
Texas (TX)	X	X	X		
Utah (UT)	X	X	X		
West Virginia (WV)	X	X	X		
Wisconsin (WI)		X	X		
Wyoming (WY)		X	X		
Total	13 States	18 States	23 States	6 States	6 States

^a^Massachusetts policies are specific to antipsychotic medications

### Specification of a Subset of Youth in Child Welfare Custody

Youth in child welfare custody may be placed in a variety of different settings, such as foster care, group homes, and residential treatment settings. Of the 48 policies enacted for youth in child welfare custody, almost half of these policies (22, 46%) applied to all youth in child welfare custody while the remainder applied to a subset of youth in child welfare custody based upon the treatment setting or placement. As noted in Table 3, 14 of the 50 states and D.C. held policies that applied to all youth in child welfare custody, whether in a state- or county-administered system.

**Table 3:** Policies enacted for informed consent of psychotropic medication use among youth in child welfare custody, by setting and consenting authority

Setting	Number of states	List of states	Number of policies
Policies enacted by setting			
Child welfare custody^a^	14	CA, FL, IL, MA, MS, NV, NC, OK, OR, PA, TX, UT, WV, WY	22
Out of home care	4	CA, FL, IL, OK	6
Psychiatric Hospital	1	IL	1
Residential Treatment	8	DE, IL, ME, ND, OR^b^, PA, TX, WV	10
Group home	7	AK, DC, NJ, NV, ND, WV, WI	7
Outpatient mental health	1	MI	1

Consenting authority^c^	Number of states	List of states	Number of policies
Policies enacted by consenting authority			
Youth^d^	8	IL, ME, NJ, OR, PA, UT, WV, WI	10
Parent	17	AK, CA, DC, DE, FL, IL, MA, ME, MI, NC, ND, OR, PA, SC, WV, WI, WY	28
Guardian	18	AK, CA, DC, DE, FL, IL, ME, MI, NJ, NC, ND, OR, PA, SC, UT, WV, WI, WY	28
Legal custodian	5	AK, IL, NC, ND, WI	7
Child welfare agency staff	8	IL, MI, MS, NC, OK, OR, PA, SC	13
Judicial system	5	CA, DC, FL, IL, MA	9
Other^e^	2	NV, TX	9

^a^In this analysis, policies applied to children/youth in child welfare custody across state or county-level contexts; only three states (CA, FL, PA) held policies that applied to specific counties across the state

^b^One state (OR) specified informed consent policy for the subset of youth in child welfare custody who were in a residential setting for individuals with intellectual disabilities

^c^While policies typically did not specify a requirement for multiple individuals to consent, policies in PA (55 Pa. Code § 5310.171) required consent from both staff at the Department of Children and Families and the parent/guardian. Policy in IL (89 Ill. Adm. Code 325 App. A 325) required consent from both the youth and the Department of Children and Family Services Guardian

^d^Age of youth consent ranged from youth over 12 years of age to youth over 15 years of age

^e^Two states did not fit into our coding scheme listed above, including NV and TX. NV was not specific in who was consenting authority but instead provided the power to “Person legally responsible for psychiatric care” of the child, TX policies delineated consenting authority to “Person legally authorized to provide consent determined by the court.”

### Gathering of Social and Medical History

A requirement for specific information to be gathered and consulted prior to the prescribing decision was specified in 19 policies across 13 states (including D.C.). Procedural elements of an informed consent process initially required information to be gathered on the medical and medication history of the child and to collect information from caregivers or other collaterals about the child’s social and/or medical history. As described in Table 4, informed consent policies in 11 states indicated the need to gather a medical history broadly defined, six states stated the need for a medication history specifically, and five states endorsed informed consent policies that emphasized the need to acquire relevant information from collaterals.

**Table 4:** Policies endorsing specific information be gathered prior to prescribing and authorizing psychotropic medications, by State

State	Regulation/Statute	Setting	Gather Information required for an informed consent		
			Medical history	Medication history	Collaterals
CA	West’s Ann.Cal.Welf. & Inst.Code § 739.5	Child Welfare custody	Yes	No	Yes
	CA Local Rules of the Tulare County Consolidated Superior Court, Rule 1108	Out of home care	No	No	Yes
DC	D.C. Mun. Regs. Tit. 29, § 6264	Group Home	No	Yes	No
FL	West’s F.S.A. § 39.407	Out of home care	Yes	No	Yes
IL	89 Ill. Adm. Code 325 App. A 325	Child Welfare custody	Yes	Yes	Yes
	89 Ill. Adm. Code 325.40	Child Welfare custody	No	Yes	No
MS	MS ADC 18-6:1.D-VII	Child Welfare custody	Yes	No	No
NJ	200.0 N.J.A.C. 3A:56-7.5	Group Home	Yes	Yes	No
NV	N.R.S. 432B.4687	Child Welfare custody	Yes	No	Yes
ND	NDAC 75-03-16-23	Residential treatment Group homes	No	Yes	No
OK	OK ADC 340:75-6-88	Out of home care	Yes	No	No
OR	OAR 413-070-0430	Child Welfare custody	Yes	Yes	No
TX	26 TAC § 749.1603	Child Welfare custody	Yes	No	No
	26 TAC § 748.2253	Residential treatment	Yes	No	No
	26 TAC § 749.1605	Child Welfare custody	Yes	No	No
	26 TAC § 748.2255	Residential treatment	Yes	No	No
	VTCA Family Code S 266.0042	Child Welfare custody	Yes	No	No
UT	U.A.C. R523-8	Child Welfare custody	Yes	Yes	Yes
WV	W. Va. Code St. § 78-3-14	Out of home care Residential treatment	Yes	No	No
Total articles of legislation endorsed			15	7	6
Total number of states with legislation endorsed			11	6	5

### Specification of Activities Required During the Clinical Encounter

Policies for informed consent may require the prescribing clinician to conduct specific actions when deciding to prescribe the medication. Our analyses of the legislation identified multiple activities that were required during the process of prescribing the psychotropic medication, including: (1) specifying the protocol for how to conduct the mental health assessment, (2) sharing information on the prescription, (3) conducting relevant laboratory tests, and (4) specifying the need and process of monitoring for potential side effects. Requirements of the clinical encounter prior to prescribing psychotropic medications were stated in 26 policies across 17 states (including D.C.).

Of the 17 states with an informed consent policy specifying procedural elements at the clinical encounter, 15 states required that information be shared regarding the prescription medication, such as requirements for administration of the prescribed medication and side effect profile. In specifying the procedural elements of prescribing, states most commonly specified provisions for a mental health assessment (n = 7), requirement to share information on the prescription (n = 15), providing laboratory tests for baseline assessments (n = 2), and specification of the need to and process for monitoring of side effects specifically (n = 6). Table 5 provides a summary of all identified policies, indicating the setting for which the policy applied, and activities required to occur during the clinical encounter.

**Table 5:** Policies endorsing specific requirements of the clinical encounter for psychotropic medication prescribing among youth in foster care, by State

State	Regulation/Statute	Setting	Requirements of clinical encounter			
			Conduct mental health assessment	Share prescription information	Provide laboratory tests	Monitor side effects
AK	7 AAC 10.1070. Medications	Group home	Not specified	Yes	Not specified	Not specified
CA	West’s Ann.Cal.Welf. & Inst.Code § 739.5	Child Welfare custody	Yes	Yes	Not specified	Yes
	Local rules of the tulare county Consolidated superior court, rule 1108	Out of home care	Yes	Yes	Not specified	Not specified
DC	D.C. Mun. Regs. Tit. 29, § 6264	Group home	Not specified	Yes	Not specified	Not specified
FL	West’s F.S.A. § 39.407	Out of home care	Yes	Yes	Not specified	Not specified
	Fla.R.Juv.P. Rule 8.355 Rule 8.355	Child Welfare custody	Yes	Not specified	Not specified	Not specified
	Fla. Admin. Code r.65C-35.001	Out of home care	Not specified	Yes	Not specified	Not specified
IL	89 Ill. Adm. Code 325 App. A 325	Child Welfare custody	Yes	Yes	Yes	Yes
ME	10-148 CMR Ch. 18-A, § 4	Residential treatment	Yes	Not specified	Not specified	Yes
MS	MS ADC 18-6:1.D-VII	Residential treatment	Not specified	Yes	Not specified	Not specified
NJ	200.0 N.J.A.C. 3A:56-7.5	Group home	Not specified	Yes	Yes	Yes
NV	NV N.R.S. 432B.4687	Child Welfare custody	Not specified	Yes	Not specified	Not specified
ND	NDAC 75-03-16-23	Residential treatment Group home	Not specified	Yes	Not specified	Not specified
OR	OAR 413-070-0430	Child Welfare custody	Yes	Not specified	Not specified	Not specified
	OR ADC 411-346-0190	Child Welfare custody	Yes	Yes	Not specified	Not specified
	OAR 411-348-0360	Residential treatment	Yes	Yes	Not specified	Not specified
PA	55 Pa. Code § 5310.171	Residential treatment	Not specified	Yes	Not specified	Yes
SC	S.C. Code of regulations R.114-593	Residential treatment	Not specified	Yes	Not specified	Not specified
TX	26 TAC § 49.1603	Child Welfare custody	Not specified	Yes	Not specified	Not specified
	26 TAC § 748.2253	Residential treatment	Not specified	Yes	Not specified	Not specified
	26 TAC § 749.1605	Child Welfare custody	Not specified	Yes	Not specified	Not specified
	26 TAC § 748.2255	Residential treatment	Not specified	Yes	Not specified	Not specified
	VTCA Family Code S 266.0042	Child Welfare custody	Not specified	Yes	Not specified	Not specified
UT	U.A.C. R523-8	Child Welfare custody	Not specified	Yes	Not specified	Not specified
WV	W. Va. Code St. § 78-3-14	Residential treatment	Not specified	Yes	Not specified	Not specified
	Code St. R. § 78-2-9 § 78-2-9	Child Welfare custody	Yes^a^	Not specified	Not specified	Not specified
	W. VA. Code St. R. § 78.3-14	Residential treatment	Not specified	Yes	Not specified	Not specified
WI	Wis. Adm. Code § DCF 57.25	Group home	Yes^b^	Not specified	Not specified	Not specified
WY	WY rules and regulations FAMS PS Ch. 3 s 16	Child Welfare custody	Not specified	Yes	Not specified	Yes
Total articles of policies endorsed			11	24	2	6
Total number of states with policies endorsed			7	15	2	6

^a^The assessment from the physician is not specific to mental health

^b^The evaluation shall be documented in the residents’ record within the first 45 days after the resident has first received a psychotropic medication

#### Vesting of Authority to Provide Informed Consent

All 23 states endorsed policies that designated an authority for consenting to psychotropic medication use among youth in child welfare custody. These policies varied in: (1) the authority who was designated, whether the youth or a surrogate decision-maker, (2) whether written documentation was required, and (3) whether youth were explicitly provided the right of refusal.

**Fig. 2:**
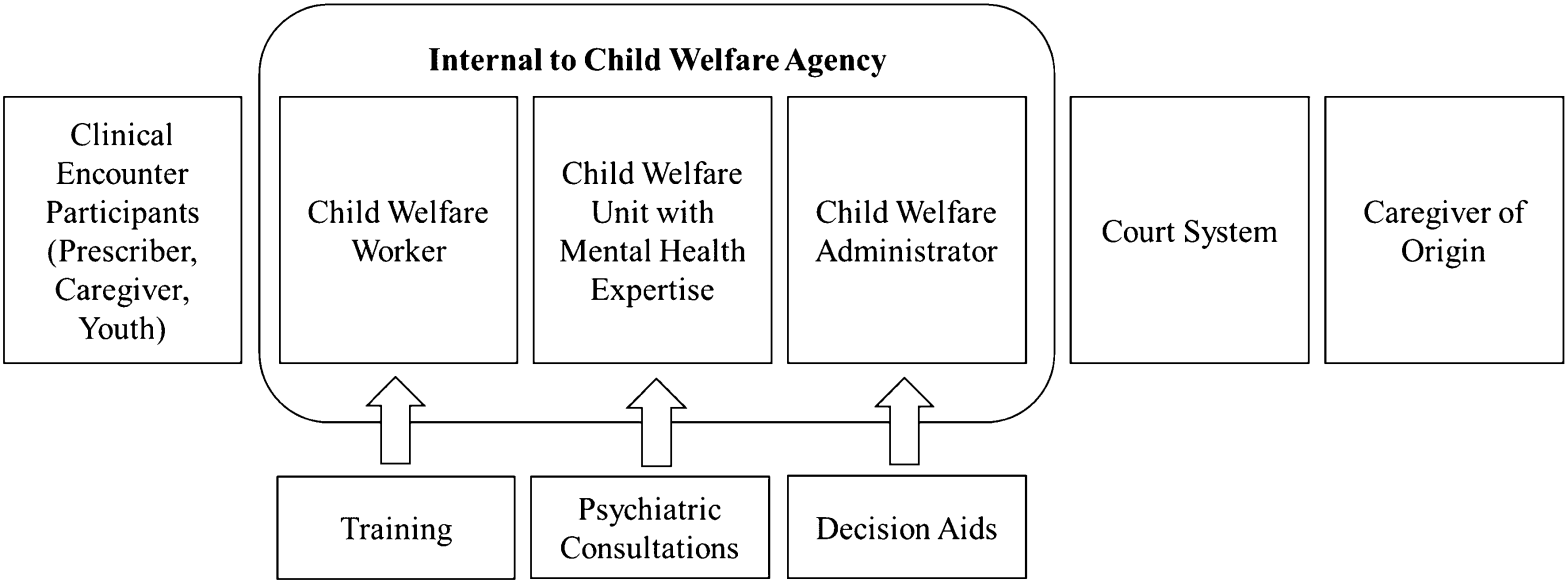
State variation in third-party vested with authority to consent to psychotropic medications prescribed to children in child welfare custody

Figure 2 illustrates the multiple types of individuals vested with the authority to consent to psychotropic medication use across all states. First, some states vested decision-making authority among the individuals present at the clinical encounter with the prescriber, caregiver, and possibly youth participating in the informed consent process during the pediatric visit. In others, decision-making authority resided within the child welfare agency whether consenting authority was vested with the child welfare caseworker, supervisor, or an individual/unit with health and/or mental health expertise. Decision-making authority also resided external to the child welfare agency (e.g., the court system). Finally, some states vested the caregiver of origin with decision-making authority until reunification no longer remained a service plan goal. Variation also existed in the extent to which training, psychiatric consultation and decision aids were made available to those with consenting authority.

States endorsed informed consent policies that indicated a distinct set of surrogate decision-makers for youth in child welfare custody. As described in Appendix Table 2, states endorsed policies articulating who was vested with consenting authority, including the guardian (n = 16), the parent (n = 15), child welfare agency staff (n = 8), legal custodian (n = 5), the judicial system (n = 5), and the youth, themselves (n = 8). Notably, policies also commonly indicated the potential for multiple individuals to consent to the use of psychotropic medications for youth in child welfare custody rather than designating authority to a single individual alone. In some cases, the conditions under which the individual could consent to psychotropic medications use were well-formulated. For example, two states (PA, IL) articulate that all individuals vested with decision-making authority were required to provide consent for psychotropic medications. In other cases, policies clearly specified the conditions upon which the designated decision-maker could address psychotropic medication use. For example, the conditions upon which youth could provide consent were generally clearly specified (e.g., specification of an age and when developmentally appropriate). In other cases, policies articulated multiple individuals could serve as a surrogate decision-maker but did not specify fully the conditions upon which this was possible. There were also two cases in which were unable to assign a designated authority. Notably, in one state, the consenting authority in the policy denoted the “person legally responsible for psychiatric care” (NV). In a second state, the legislation identified a process by which courts would identify the surrogate decision-maker (TX). In these cases, the lack of specificity (NV) and potential variation of the designated authority (TX) prevented our ability to code the consenting authorities in the taxonomy.

As detailed in Table 6, policies also indicated, to varying extent, whether youth in child welfare custody held assenting authority for psychotropic medication use. Six states held policies that specified the right for all youth in child welfare custody to refuse psychotropic medication use. States also endorsed policies explicitly recognizing youth assent for psychotropic medication prescribing in specific contexts, specifically group homes (n = 3), residential treatment (n = 3) and out-of-home care (n = 1).

**Table 6:** Policies endorsing specific requirements for authorizing, notifying, and reviewing psychotropic medication use for youth in child welfare custody, by State

State	Regulations/Statutes	Setting	Authorize, Right to Refuse^a^	Notification, parties notified^b^	Review of prior decision, time specified^c^
AK	7 AAC 10.1070. Medications	Group home	Yes	Not specified	Not specified
CA	Rule 5.640. Psychotropic medications	Child welfare custody	Not specified	Caseworker, Court Appointed Special Advocate, Group home, if applicable	At least every 6 months, review to occur at regularly scheduled court hearing
	West’s Ann.Cal.Welf. & Inst.Code § 739.5	Child welfare custody	Not specified	Not specified	Review to occur at regularly scheduled court hearings and reports provided by the county probation agency
DE	Del. Admin. Code 105-3.0	Residential treatment	Not specified	Not specified	Monthly
FL	West’s F.S.A. § 39.407	Out of home care	Yes	All appropriate parties	Not specified
	Fla.R.Juv.P. Rule 8.355 Rule 8.355	Child welfare custody	Yes	All appropriate parties and child’s attorney	Not specified
	Fla. Admin. Code r. 65C-35.007	Out of home care	Yes	Not specified	Not specified
IL	89 Ill. Adm. Code 325 App. A 325	Child welfare custody	Yes	Not specified	Annually
	89 Ill. Adm. Code 325.40	Child Welfare custody	Yes	Not specified	Annually
	89 Ill. Adm. Code 325.50	Residential treatment	Not specified	Not specified	Annually
	89 Ill. Adm. Code 325.60	Psychiatric hospitals	Not specified	Not specified	Annually
ME	10-148 CMR Ch. 18-A, § 4	Residential treatment	Yes	Not specified	Monthly
MS	MS ADC 18-6:1.D-VII	Child welfare custody	Not specified	Parent or guardian	Not specified
NC	N.C.G.S.A. § 7B-505.1	Child welfare custody	Not specified	Parent, guardian or custodian	Not specified
NJ	200.0 N.J.A.C. 3A:56-7.5	Group home	Yes	Not specified	Not specified
NV	N.R.S. 432B.4688	Child welfare custody	Not specified	Not specified	Quarterly
OK	OK ADC 340:75-6-88	Out of home care	Not specified	Child’s parent or legal guardian	Not specified
	OKLa. Admin. Code 340:75-14-3	Child welfare custody	Not specified	Child’s parent or legal guardian	Not specified
OR	OAR 413-070-0430	Child welfare custody	Not specified	Caseworker charged to notify stakeholders	Annually, if (a) child has more than two prescriptions for psychotropic medications or (b) child under 6 years of age
	OR ADC 411-346-0190	Child welfare custody	Not specified	Child Welfare if state is not the legal guardian	Not specified
	OAR 411-348-0360	Residential treatment	Not specified	Service coordinator and parent or legal guardian	Not specified
PA	55 Pa. Code § 3680.52	Child welfare custody	Yes	Not specified	Not specified
	55 Pa. Code § 3130.91	Child welfare custody	Yes	Not specified	Not specified
SC	S.C. Code of Regulations R. 114-593	Residential treatment	Yes	Not specified	Not specified
TX	26 TAC § 749.1603	Child welfare custody	Yes	Not specified	Not specified
	26 TAC § 748.2253	Residential treatment	Yes	Not specified	Not specified
	26 TAC § 749.1605	Child welfare custody	Yes	Not specified	Not specified
	26 TAC § 748.2255	Residential treatment	Yes	Not specified	Not specified
UT	U.A.C. R523-8	Child welfare custody	Yes	Not specified	Not specified
WV	Code St. R. § 78-2-9 § 78-2-9	Child welfare custody	Yes	Not specified	Not specified
WI	Wis. Adm. Code § DCF 57.25	Group home	Yes	Not specified	Not specified
Total articles of policies endorsed			18	10	10
Total number of states with policies endorsed			11	6	6

^a^Policies endorsing specific information be gathered prior to prescribing and authorizing psychotropic medications, by State

^b^State policies endorsing the right to refuse

^c^Policies that endorse notifying specific stakeholder of decision to prescribe psychotropic medication for youth in child welfare custody

Supplemental analyses also investigated the number of procedural elements endorsed based on the consenting authority of the respective state policy. Analyses found minimal variation in the average number of procedural elements endorsed across consenting authorities, ranging from on average 2.6 procedural elements for states delegating consent to legal custodians to an average of 3.5 procedural elements for states delegating consenting authority to individuals not otherwise captured in our categorical list. (See Appendix Table 3.)

### Notification of Consenting Decision to Stakeholders

Policies may also require that specific stakeholder be notified of the decision to authorize psychotropic medications. Requirements that those specific stakeholders were notified of the prescription were stated in ten policies across six states (including DC). As articulated in Table 6, policies ranged in both the specificity and breadth of individuals for whom notification was indicated, ranging from “all appropriate parties” to individuals specific to the child welfare system (e.g., Court Appointed Special Advocate, caseworker) to the parent, guardian, or the custodian.

### Ongoing Review of Consent over Time

Specification that the consenting authority revisit the prior decision for authorization within a specified timeframe or under specific circumstances was present in nine policies across five states. Four of these five states had a policy for ongoing review that pertained to all youth in child welfare custody. Two states had policies that applied to those youth in child welfare custody who were in residential treatment settings and one state held a policy for ongoing review specific to psychiatric hospitals. As described in Table 6, these policies specified both timeframes and circumstances. The majority of policies (n = 8) indicated a timeframe for ongoing review, ranging from monthly (n = 1) to annually (n = 6). Of note, one state (OR) conditioned an ongoing annual review on specific prescribing practices, specifically highlighting the need for annual review when a child is prescribed two or more psychotropic medications concurrently or the child is under 6 years of age.

## Discussion

This article identifies a taxonomy for the procedural elements of informed consent based upon existing child welfare policy and then assesses the extent to which these are codified in statutes or regulations that are publicly accessible. Specification of these procedural elements in formal policies is a critical aspect of promoting transparency and facilitating public discourse and legal protections for vulnerable populations, such as youth in child welfare custody (Noonan & Miller, 2013).

Drawing on key informant interviews with child welfare mid-level administrators, this study defines five procedural elements of existing policies governing informed consent for the administration of psychotropic medications to youth in child welfare custody, including: (1) information to be gathered about the child’s social and medical history, (2) activities required to inform the prescribing decision, (3) the individual vested with authority to consent to psychotropic medication use, (4) the process for notifying stakeholders of the prescription, and (5) parameters for ongoing review of the prior decision. Our review identified 23 states with legislation detailing some part of the informed consent process, but only two states specified all five of the procedural elements identified in the taxonomy. Moreover, the content of each procedural element varied across policies. For instance, while all states delineated the individual(s) authorized to consent (whether the youth or a surrogate decision-maker), variation existed to whom that responsibility was assigned across the states. Thus, while our taxonomy provides a framework for understanding and potentially creating policies around informed consent, there is still much to be understood about whether, how, and why each of these procedural elements for informed consent are specified and in what contexts.

The procedural elements of informed consent set requirements *prior to* the clinical encounter, *during* that encounter, and *after* the decision to authorize psychotropic medication use. That is, policies that include procedural elements prior to and following the consenting decision itself. This suggests an informed consent process requires an ongoing commitment, relying on information prior to the consenting decision, itself, and the ability to ensure ongoing review after the consenting decision has been made. Professional standards of care for youth in child welfare custody emphasize the need to gather both medical and social histories of youth in child welfare custody before providing medical and mental health care (Substance Abuse and Mental Health Services Administration, 2018). Consistent with these professional recommendations, our study reveals that formal policies do, in some cases, articulate the collection of social and medical history *before* the consenting decision occurs. Formal policies may also require revisiting the decision of consent throughout the duration of caring for the patient, which is consistent with professional standards for ongoing oversight of prescribed psychotropic medications. The need for ongoing monitoring warrants particular consideration given the limited data on long-term efficacy and potential side effects of psychotropic medication use among children and adolescents (Quinlan-Davidson et al., 2021). However, the absence of these procedural elements in many of the formal policies endorsing informed consent present represents a failure to leverage the potential of formal policies to facilitate compliance and accountability with standards of care (Noonan & Miller, 2013; Raghavan et al., 2008).

At the same time, the presented taxonomy of procedural elements in informed consent policies also fails to respond fully to calls for shared decision-making. Our taxonomy sought to capture procedural elements of informed consent policies in practice rather than in an ideal world. Determination of the latter is a critical next step for this work. For example, opportunities exist for the procedural elements of an informed consent process to highlight the agency and involvement of youth in every step of the process. Multiple stakeholders have articulated the necessity for youth to be provided with agency throughout the informed consent process and to hold decision-making authority for themselves, whenever possible (Simmel et al., 2021). Additionally, the quality of engagement could be more deeply conceptualized to facilitate multi-directional communication and education. For example, the final step of notification could be articulated as education to ensure that all relevant individuals, including the youth themselves, are not only notified but also educated about the treatment provided. Future work and expert consensus with youth in child welfare custody or alumni of the foster care system, caregivers of origin, caseworkers, foster parents, clinicians, among others is warranted to provide additional specification of a “patient- and stakeholder-centered” taxonomy for the procedural elements of an informed consent process for psychotropic medications for youth in child welfare custody. Such a taxonomy could be compared to the taxonomy presented in this paper to identify opportunities for greater alignment and improvement in current child welfare policy and practice.

All five procedural elements were not uniformly applied across states. The decisions at the state level to include or conversely *not to* include an element likely reflects legislators conceptualization of the goals and purposes of informed consent. While the child welfare system is charged with the three goals of safety, permanency, and well-being in the Adoption and Safe Families Act (1997), decisions about the informed consent process and surrogate decision-maker may reflect which of these goals is prioritized. For instance, states that include judicial oversight of prescribing decisions may be prioritizing the goal of safety and well-being while clinical review may be placing priority on the medical expertise specifically. In contrast, designation of decisional authority to the caregiver of origin demonstrates a commitment of the system to the goal of reunification and permanency. The variability identified in formal policies endorsing informed consent creates opportunities for confusion or gaps that may generate arbitrary decision-making. The variability in the presence and content of the procedural elements included in these formal policies is worth particular mention. For example, a prior systematic review emphasizes that youth in child welfare custody report experiencing inadequate engagement in the discussion of treatment alternatives and potential side effects (Barnett et al., 2019). Specification of these procedures at the time of prescribing in formal policies increases the potential not only for standardization but also legal protection.

Our findings also suggest that a single state may hold multiple formal policies in place to specify informed consent for psychotropic medication use among youth in child welfare custody. These policies specified different procedural elements of the informed consent process or specific sub-populations for which the policy articulated a specific process. This variation, on the one hand, provides opportunities for procedural elements to be integrated in other policy domains with valuable adaptations being provided to accommodate the wide array of placement settings youth in child welfare custody may have. At the same time, variation in the formal policies, if not clearly operationalized for child welfare and youth-serving partners, may create the possibility of confusion around the requirements to individuals (e.g., caseworkers, out-of-home treatment providers) critical in implementing the informed consent policy for youth in child welfare custody (Lipsky, 2010). Implementation of these formal policies fundamentally rely on a diverse array of individuals to implement them and their understanding and perception of formal policies regarding informed consent is a critical area to identify and address potential policy implementation challenges.

These findings could be used for policymakers to consider whether their policies align with the procedural elements endorsed in other child welfare settings. For instance, policymakers could examine these five elements when designing a policy to determine which are applicable to their particular case. Such guidance could assist policymakers in arriving at consensus on the procedural elements to include in development of their respective policies. Some elements specified for youth in child welfare custody may reflect the particular challenges that characterized informed decision-making. For example, the need to notify various individuals of the decision to prescribe psychotropic medications for youth in child welfare custody is especially important, given the number of individuals that are involved in the life of the youth and the transitions routinely experienced by these youth. In other cases, specification of the treatment administration, risks and benefits, and side effect protocols of a proposed treatment applies broadly for medical care and is consistent with standards of patient-centered care. At the same time, critical to design of informed consent policy would also be integration of the perspective of youth and other relevant community members from within their jurisdiction (e.g., caregivers, biological parents, caseworkers).

Notably, the National Academy of Medicine among others has made calls to encourage dialogue between physicians and patients in the decision-making process (Committee on Quality of Health Care in America & Institute of Medicine, 2001). These calls emphasize that patient-centered care, which is both respectful and responsive to the preferences, needs and values of the patient and their caregiver, should guide clinical decisions. As a principle of medical practice, patient-centered care emphasizes the need for dialogue around the relative merits of potential treatment alternatives in all medical care. Specific procedural elements, such as sharing information on the medication itself and providing youth the ability to consent or assent for the medications themselves, emphasize key elements of patient-centered care. However, other aspects of the policies identified, such as reliance on a consenting authority not at the clinical encounter, may challenge whether shared decision-making can occur without efforts to ensure responsiveness to the needs and values of the patient and their caregiver.

In addition, further examination of the policy-making process could help us understand how legislators consider the trade-offs that present themselves in specifying procedural elements of informed consent in policy. On the one hand, formal policy presents the benefit of generating transparency with regard to required procedural elements for informed consent providing opportunity for accountability in prescribing psychotropic medications. On the other hand, formal policy may present liabilities for the state system should they not comply with regulations. Obtaining procedural elements of informed consent may also provide the decisional support that caregivers of youth in child welfare custody indicate needing (Barnett et al., 2016a). For example, prior research suggests caregivers rely heavily on finding information about prescribed psychotropic medications themselves (Barnett et al., 2016b). Moreover, stakeholders including caregivers and caseworkers have highlighted the importance of those who know the child best centrally involved in decision-making; participation in clinical visits; the problems of delays in important treatment decisions when individuals authorized to consent are not immediately available; and the value of shared decision-making in an inclusive team approach (Simmel et al., 2021). On the other hand, stakeholders have also identified the potential value of a single external authority to provide objective oversight and guard against the hazards of overprescribing, inappropriate dosages, and polypharmacy (Simmel et al., 2021). These considerations highlight the complex trade-offs involved in achieving a process that achieves the tenets of shared decision-making with the desire to centralize decision-making authority with individuals offering the clinical expertise required to provide oversight of medication decisions. Future research is also needed to identify whether enactment of informed consent policies is effective in addressing concerns regarding safe and judicious use; no such studies were identified in a relevant systematic review of strategies to advance the safe and judicious use of antipsychotic medications (Mackie et al., 2020a, 2020b). Whether through formal or informal policies, opportunities exist for legislators and child welfare administrators to consider the commitments they hold to ensuring decisions that are both well-informed and in the best interests of the child.

Additionally, investigations into the factors influential to the development of and variation among state informed consent policies presents an important area for additional inquiry. Despite federal calls and coordinated technical assistance efforts to facilitate psychotropic medication oversight among youth in child welfare custody (Mackie et al., 2017, Substance Abuse and Mental Health Services Administration, 2018), substantial variation persists in the extent of state policy endorsement nationally. While prior research examines how states engaged evidence in “defining the problem of psychotropic medication use” (Hyde et al., 2016), additional research is warranted to investigate how informed consent policies emerged in response to these concerns and the role of evidence and relevant stakeholders in agenda setting and policy formulation. Such inquiry would provide important opportunity to identify potential opportunities to advance development of state policies that facilitate both transparency and accountability in the informed consent process implemented by child welfare agencies.

## Limitations

Our review is a first step in the process of creating additional transparency in procedural elements currently endorsed by state legislation. The taxonomy, itself, represents the procedural elements of informed consent policies as articulated by mid-level administrators in public sector agencies. The data presented in this article do not reflect the perspectives from key stakeholders, including youth in or alumni of the child welfare system. Given the well-documented challenges to processes of meaningful informed consent and shared decision-making in child welfare settings, a critical next line of inquiry is whether these procedural elements adequately respond to the needs of youth and other key community partners. Opportunities also exist to ensure the procedural elements articulated in this paper are consistent with current practice. Therefore, future research is needed to determine whether procedural elements identified are consistent with contemporary child welfare practice and align with the priorities of key stakeholders, including youth.

Our initial set of interviews with mid-level administrators to establish the procedural elements of child welfare policies occurred in 2009–2010. This framework presented was triangulated methodologically with the legislative review conducted with data arriving from as recent as February 2022. The taxonomy developed in 2009–2010 served as a helpful framework to the organization of the data collated through the legislative review. Future research might investigate whether recent informal policies require an update and modification to the taxonomy of procedural elements endorsed by child welfare agencies.

Additionally, our review of statutes and regulations captures only a subset of the policies that may be endorsed by state or county child welfare agencies. Agencies may hold policies for informed consent that were implemented organizationally but are not legislatively mandated. It is also important to note that our analyses only sought to identify policies that specifically sought to indicate provisions for youth in child welfare custody; other policies may exist generally for youth placed in a variety of different settings. If these policies did not provide a specific provision for youth in child welfare custody, then we did not include these policies in the present analyses. Future research will be important to consider the informed consent policies in place more broadly for youth engaged in specific treatment settings, such as residential treatment settings, psychiatric hospitals, etc.

## Conclusions

The present study identifies a taxonomy for the procedural elements of child welfare policies that endorse an informed consent process for psychotropic medications among youth in child welfare custody. The article subsequently identifies whether formal legislative policies across the 50 states and D.C. endorse these five procedural elements and characterizes the variation that exists. Only two states endorsed policies that included all five procedural elements and drastic variation characterized the content of these policies. This paper argues that additional specification in informed consent policies is needed to advance the opportunity for policy to promote professional standards of care and best practice. Opportunities also exist for future studies to consider the applicability of this framework in the context of other cases of informed consent, especially in cases of surrogate decision-making.

## Supplementary Information

Below is the link to the electronic supplementary material.

Supplementary file1 (DOCX 33 KB)
