# Supplementary material for: The Role of Formal Policy to Promote Informed Consent of Psychotropic Medications for Youth in Child Welfare Custody: A National Examination
Source: Adm Policy Ment Health. 2022 Aug 6;49(6):986–1003. doi: 10.1007/s10488-022-01212-3 (PMC9616785; doi:10.1007/s10488-022-01212-3)
Supplement: Supplementary file 1 — Supplementary file1 (DOCX 33 KB) [file 10488_2022_1212_MOESM1_ESM.docx]

**Appendix:**

**Appendix Table 1.** *Search Terms Employed for Extraction of Legislation*

| **Domain** | **Search Term** |
| --- | --- |
| Psychotropic medications | “psychotropic” or “antipsychotic” or “psychoactive” or “psychotherapeutic” or “psychopharmacology” |
| Informed consent and assent | “informed consent” or “assent” or “consent” or “shared decision making” or “permission” |
| Children and/or youth | “children” or “youth” or “adolescent” or “infant” |
| Foster care and/or child welfare | “foster care” or “child welfare” or “child protective services” or “custody” |
| Targeted Advanced Search: adv: “psychotropic antipsychotic psychoactive psychotherapeutic psychopharmacology & informed consent assent shared decision-making permission & child! youth adolescent! infant! & foster care child welfare child protective services custody” | |

| **Appendix Table 2.** *Consenting Authority Designated in Informed Consent Policy(ies), by State* | | | | | | | | | | |
| --- | --- | --- | --- | --- | --- | --- | --- | --- | --- | --- |
| **State** | **Regulations/ Statutes** | **Setting** | **Required Documentation** | **Consenting Authority** | | | | | | |
|  |  |  |  | Youth (Age) | Parent | Guardian | Legal custodian | Child Welfare Agency Staff | Judicial System | Notes |
| AK | 7 AAC 10.1070. Medications | Group home | Written | No | Yes | Yes | Yes | No | No |  |
| CA | Rule 5.640. Psychotropic medications | Child Welfare custody | Psychotropic medication form, physician statement, proof of notice application | No | No | No | No | No | Yes |  |
|  | County of Santa Clara, Local Rules of Court, Juv. Rule 1 Rule 1. General Provisions | Child Welfare custody | Not specified | No | Yes | Yes | No | No | Yes | Judicial system if ward of the state |
|  | West’s Ann.Cal.Welf. & Inst.Code § 739.5 | Child Welfare custody | Court authorization | No | No | No | No | No | Yes |  |
|  | Local Rules of the Tulare County Consolidated Superior Court, Rule 1108 | Out of home care | Judicial council form | No | Yes | Yes | No | No | Yes |  |
| DE | Del. Admin. Code 105-3.0 | Residential treatment | Written | No | Yes | Yes | No | No | No |  |
| DC | D.C. Mun. Regs. Tit. 29, § 6264 | Group home | Parent/ Guardian: Written  Court: Court order | No | Yes | Yes | No | No | Yes |  |
| FL | West's F.S.A. § 39.407 | Out of home care | Parents: not specified  Court: Court order | No | Yes | Yes | No | No | Yes | Judicial system consent if parental rights have been terminated |
|  | Fla.R.Juv.P. Rule 8.355  Rule 8.355 | Child Welfare custody | Parent: Not specified  Court: Court order | No | Yes | Yes | No | No | Yes |  |
|  | Fla. Admin. Code r. 65C-35.001 | Out of home care | Not specified | No | Yes | Yes | No | No | No |  |
|  | Fla. Admin. Code r. 65C-35.007 | Out of home care | Parent or legal guardian: Not specified  Case Manager: | No | Yes | Yes | No | No | Yes, if parents or guardian do not consent |  |
| IL | 89 Ill. Adm. Code 401.410 | Child Welfare custody | Written | No | Yes | Yes | Yes | No | No |  |
|  | 89 Ill. Adm. Code 402.25 | Child Welfare custody | Written | No | Yes | Yes | Yes | No | No |  |
|  | 89 Ill. Adm. Code 325 App. A 325 | Child Welfare custody | DCFS: Written  Child: Verbal | Yes | No | No | No | Yes | No | DCFS Guardian and child consent required |
|  | 89 Ill. Adm. Code 325.40 | Child Welfare custody | Centralized consent unit | No | No | No | No | Yes | No |  |
|  | 89 Ill. Adm. Code 325.50 | Residential treatment | Centralized consent unit | No | Yes | Yes | No | No | No |  |
|  | 89 Ill. Adm. Code 325.60 | Psychiatric hospital | Centralized consent unit | No | No | No | No | Yes | No | DCFS Guardian |
| MA | 110 CMR 11.14 | Child Welfare custody | Written | No | Yes | No | No | No | Yes |  |
| ME | 10-148 CMR Ch. 18-A, § 4 | Residential treatment | Written | Yes | Yes | Yes | No | No | No | Youth over 14 |
| MI | MCLA 330.1707 | Outpatient mental health | Not specified | No | Yes | Yes | No | Yes | No |  |
| MS | MS ADC 18-6:1.D-VII | Child Welfare custody | Consent form | No | No | No | No | Yes | No |  |
| NJ | 200.0 N.J.A.C. 3A:56-7.5 | Group home | Written | Yes | No | Yes | No | No | No | Youth over age 14 |
| NV | NAC 424.722 | Group home | Written | ^a^ | ^a^ | ^a^ | ^a^ | ^a^ | ^a^ | Consenting authority is adjudicated by the court and will be determined to be person legally responsible for psychiatric care |
|  | N.R.S. 432B.4687 | Child Welfare custody | Written | ^a^ | ^a^ | ^a^ | ^a^ | ^a^ | ^a^ |  |
|  | N.R.S. 432B.4688 | Child Welfare custody | Not specified | ^a^ | ^a^ | ^a^ | ^a^ | ^a^ | ^a^ |  |
| NC | N.C.G.S.A. § 7B-505.1 | Child Welfare custody | Not specified | No | Yes | Yes | Yes | Yes | No |  |
| ND | NDAC 75-03-16-23 | Residential facilities  Group homes | Written | No | Yes | Yes | Yes |  | No |  |
| OK | OK ADC 340:75-6-88 | Out of home care | Not specified | No | No | No | No | Yes | No |  |
|  | Okla. Admin. Code 340:75-14-3 | Child Welfare Custody | Not specified | No | No | No | No | Yes | No |  |
| OR | OAR 413-070-0430 | Child Welfare custody | Written | Yes | Yes | Yes | No | Yes | No | Youth over age 15 |
|  | OR ADC 411-346-0190 | Child Welfare custody | Written | No | No | No | No | No | No |  |
|  | OAR 411-348-0360 | Residential treatment | Written | No | No | No | No | Yes, when CPS is the child’s guardian | No |  |
| PA | 55 Pa. Code § 5310.171 | Residential care | Written | No | Yes | Yes | No | Yes | No | Must have consent from both parent/ guardian and Child welfare |
|  | 55 Pa. Code § 3680.52 | Child Welfare custody | Written | Yes | Yes | Yes | No | No | No | Youth over age 14 |
|  | 55 Pa. Code § 3130.91 | Child Welfare custody | Written | Yes | Yes | Yes | No | No | No | Youth over age 14 |
| SC | S.C. Code of Regulations R. 114-593 | Residential treatment | Written | No | Yes | Yes | Yes | No | No |  |
| TX | 26 TAC § 749.1603 | Child Welfare custody | Written | Y/N | Y/N | Y/N | Y/N | Y/N | Y/N | Person legally authorized to provide consent determined by court |
|  | 26 TAC § 748.2253 | Residential treatment | Written | Y/N | Y/N | Y/N | Y/N | Y/N | Y/N |  |
|  | 26 TAC § 749.1605 | Child Welfare custody | Written | Y/N | Y/N | Y/N | Y/N | Y/N | Y/N |  |
|  | 26 TAC § 748.2255 | Residential treatment | Written | Y/N | Y/N | Y/N | Y/N | Y/N | Y/N |  |
|  | VTCA Family Code S 266.0042 | Child Welfare custody | Not specified | Y/N | Y/N | Y/N | Y/N | Y/N | Y/N |  |
|  | V.T.C.A., Family Code § 266.004 | Child Welfare custody | Not specified | Y/N | Y/N | Y/N | Y/N | Y/N | Y/N |  |
| UT | U.A.C. R523-8 | Child Welfare custody | Not specified | Yes | No | Yes | No | No | No |  |
| WV | W. Va. Code St.  § 78-3-14. | Residential treatment  Group home | Written | Yes | Yes | Yes | No | No | No | Youth over age 12 |
|  | Code St. R. § 78-2-9  § 78-2-9 | Child Welfare custody | Not specified | Yes | Yes | Yes | No | No | No | Youth over age 14 |
|  | W. Va. Code St. R. § 78-3-14 | Residential treatment | written | No | Yes | Yes | No | No | No |  |
| WI | Wis. Adm. Code § DCF 57.25 | Group home | Consent form | Yes | Yes | Yes | Yes | No | No | Youth over age 14 |
| WY | Rules and Regulations FAMS PS Ch. 3 s 16 | Child Welfare custody | Consent form | No | Yes | Yes | No | No | No |  |
| ^a^Policy did not articulate the specific role of the individual designated to provide consenting authority. | | | | | | | | | | |

**Appendix Table 3.** *Procedural Elements by Consenting Authority*

| **Consenting Authority** | **Number of States** | **List of States** | **Average Number of Procedural Elements Endorsed** | **Range of Number of Procedural Elements Endorsed** |
| --- | --- | --- | --- | --- |
| Youth | 8 | IL, ME, NJ, OR, PA, UT, WV, WI | 3 | 2-4 |
| Parent | 17 | AK, CA, DC, DE, FL, IL, MA, ME, MI, NC, ND, OR, PA, SC, WV, WI, WY | 2.7 | 1-5 |
| Guardian | 18 | AK, CA, DC, DE, FL, IL, ME, MI, NJ, NC, ND, OR, PA, SC, UT, WV, WI, WY | 2.8 | 1-5 |
| Legal Custodian | 5 | AK, IL, NC, ND, WI | 2.6 | 2-4 |
| Child Welfare Agency Staff | 8 | IL, MI, MS, NC, OK, OR, PA, SC | 2.75 | 1-4 |
| Judicial System | 5 | CA, DC, FL, IL, MA | 3.4 | 1-5 |
| Other | 2 | NV, TX | 3.5 | 3-4 |
